# Effect of perioperative oral care on prevention of postoperative pneumonia associated with esophageal cancer surgery: A multicenter case–control study with propensity score matching analysis

**DOI:** 10.1097/MD.0000000000007436

**Published:** 2017-08-18

**Authors:** Sakiko Soutome, Souichi Yanamoto, Madoka Funahara, Takumi Hasegawa, Takahide Komori, Shin-ichi Yamada, Hiroshi Kurita, Chika Yamauchi, Yasuyuki Shibuya, Yuka Kojima, Hirokazu Nakahara, Takahiko Oho, Masahiro Umeda

**Affiliations:** a Perioperative Oral Management Center, Nagasaki University Hospital; b Department of Clinical Oral Oncology, Nagasaki University Graduate School of Biomedical Sciences, Nagasaki; c Department of Oral and Maxillofacial Surgery, Kobe University Graduate School of Medicine, Kobe; d Department of Dentistry and Oral Surgery, Shinshu University School of Medicine, Matsumoto; e Department of Oral Maxillofacial Surgery, Graduate School of Medical Sciences, Nagoya City University, Nagoya; f Department of Dentistry and Oral Surgery, Kansai Medical University, Hirakata; g Department of Oral and Maxillofacial Surgery, Osaka City University Graduate School of Medicine, Osaka; h Department of Preventive Dentistry, Kagoshima University Graduate School of Medical and Dental Sciences, Kagoshima, Japan.

**Keywords:** esophageal cancer surgery, oral care, postoperative pneumonia, propensity score

## Abstract

The aim of this study was to investigate the effectiveness of oral care in prevention of postoperative pneumonia associated with esophageal cancer surgery.

Postoperative pneumonia is a severe adverse event associated with esophageal cancer surgery. It is thought to be caused by aspiration of oropharyngeal fluid containing pathogens. However, the relationship between oral health status and postoperative pneumonia has not been well investigated.

This study included 539 patients with esophageal cancer undergoing surgery at 1 of 7 university hospitals. While 306 patients received perioperative oral care, 233 did not. Various clinical factors as well as occurrence of postoperative pneumonia were retrospectively evaluated. Propensity-score matching was performed to minimize selection biases associated with comparison of retrospective data between the oral care and control groups. Factors related to postoperative pneumonia were analyzed by logistic regression analysis.

Of the original 539 patients, 103 (19.1%) experienced postoperative pneumonia. The results of multivariate analysis of the 420 propensity score-matched patients revealed longer operation time, postoperative dysphagia, and lack of oral care intervention to be significantly correlated with postoperative pneumonia.

The present findings demonstrate that perioperative oral care can reduce the risk of postoperative pneumonia in patients undergoing esophageal cancer surgery.

## 1 Introduction

With the development of various treatment methods, including perioperative management, prognosis in patients with esophageal cancer has improved in recent decades.^[1]^ However, some adverse events, such as suture insufficiency and cardiovascular and respiratory complications, often occur postoperatively.^[2]^ Postoperative pneumonia is one of the more frequent and possibly fatal complications among patients who undergo major esophageal surgery. In a study by Ando et al,^[3]^ the rate of incidence of respiratory complications was 19.5%, and severity of postoperative complications, degree of residual tumor, and number of dissected mediastinal nodes were found to be significant prognostic factors among patients who underwent esophageal cancer surgery. The authors also stated that approximately half the cases of early death after surgery for advanced esophageal cancer were caused by pulmonary complications. Many authors have reported risk factors for postoperative pneumonia after esophageal resection; factors such as old age, pulmonary function, diabetes mellitus, and surgical stress were found to be related to the frequency of postoperative pneumonia,^[4–18]^ although the relationship between oral health status and postoperative pneumonia was not described in these studies.

One of the main causes of postoperative pneumonia is thought to be aspiration of oropharyngeal fluid containing pathogenic microorganisms.^[19]^ Akutsu et al^[20]^ described that tooth brushing by the patients 5 times per day decreased the incidence of postoperative pneumonia in patients undergoing esophagectomy with thoracotomy. Hiramatsu et al^[21]^ also reported that preoperative professional oral cleaning and teeth and tongue brushing with deep breathing, breathing exercises, respiratory muscle stretching, proper diet, and cessation of smoking prevented postoperative pneumonia in those who underwent esophageal cancer resection. However, those studies were based on a small number of patients with historical control, and did not analyze other risk factors for postoperative pneumonia. There have been no well-designed studies that show the effects of oral health care on prevention of postoperative pneumonia after esophageal cancer surgery. In a previous retrospective study involving a relatively small number of patients, we, too, have reported that perioperative oral care might reduce the risk of postoperative pneumonia after esophageal resection.^[22]^ The purpose of this study is to investigate the effectiveness of perioperative oral care in prevention of postoperative pneumonia by multicenter retrospective analysis of a large number of patients using propensity score matching analysis.

## 2 Methods

### 2.1 Study design

This study was a multicenter, case–control study with propensity score matching analysis.

### 2.2 Patients

**Figure 1:**
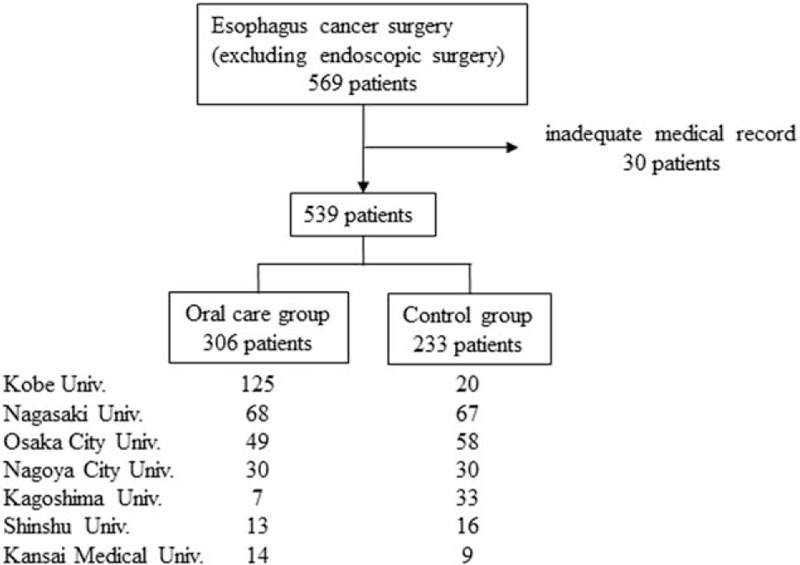
Patients distribution to the oral care and control groups in each hospital.

Between 2011 and 2015, 569 patients with esophageal cancer underwent surgery, excluding endoscopic mucosal resection or submucosal dissection, at 1 of 7 university hospitals, including those of Nagasaki University, Kobe University, Kagoshima University, Kansai Medical University, Nagoya City University, Shinshu University, and Osaka City University. Among them, 30 patients with inadequate medical records were excluded, and the remaining 539 patients were included in the study. The included patients were divided into 2 groups: an oral care group (n = 306) and a control group (n = 233 patients). In Japan, the Medical Insurance System established coverage for perioperative oral care in 2012. Patients in the control group had undergone surgery before the start of perioperative oral care in each hospital, while those in the oral care group had undergone surgery after the said period. Since the time of introduction of oral care depending on the hospital, the rate of patients who received oral care differs (Fig. 1).

### 2.3 Oral care intervention

Each patient in the oral care group received oral care from a dentist and dental hygienist. Oral care was started from the time the decision for hospitalization was made. It included oral health instruction, removal of dental calculus (scaling), professional mechanical tooth cleaning (PMTC), removal of tongue coating with a toothbrush, cleaning denture, and extraction of teeth with severe periodontitis showing pain, pus discharge, mobility, or marked alveolar bone loss by X-ray examination. Patients were instructed to clean teeth by toothbrush, interdental brush, dental floss, followed by gargling 3 times per day. PMTC was performed by the method reported by Axelsson and Lindhe,^[23]^ including polishing using rubber cup, brush, and rubber tip with polishing paste containing fluoride. Edenturous patients received only cleaning of the tongue and denture, and instruction to gargling. All patients received final oral cleaning by a dentist or dental hygienist the day before surgery. After surgery, patients of both groups were asked to perform frequent (every 3–6 hours) gargling with water during the daytime.

### 2.4 Variables

The objective variable was occurrence of postoperative pneumonia. Pneumonia was diagnosed by the presence of fever, elevated white blood cell and C-reactive protein (CRP) levels, and pulmonary infiltrates requiring antibiotic therapy, according to the criteria reported by several investigators.^[2,4,11]^ On the basis of previous literature,^[2–16]^ predictor variables were defined as patient factors: age, sex, body mass index (BMI), smoking and drinking habits, diabetes mellitus, hypertension, preoperative serum creatinine and albumin concentrations, and forced expiratory volume (FEV) 1%; tumor factors: site and stage; treatment factors: operation method (open thoracic esophagectomy or thoracoscopy-assisted esophagectomy), operation time, blood loss, and neoadjuvant chemotherapy; postoperative dysphagia; and oral care intervention. Patients who had not smoked for a year or longer were classified as not having a smoking habit. Tumors were categorized as being located in the upper, middle, or lower esophagus. Tumor stage was classified according to the criteria proposed by the Japan Esophageal Society.^[24]^ Postoperative dysphagia was defined on the basis of medical records as choking when the patient started a paste diet after surgery, aspiration during swallowing (observed through videofluoroscopic examination), or continued tube feeding at the time of discharge.

Further, duration of hospitalization and death within 30 days of hospitalization were compared between the oral care and control groups.

### 2.5 Statistical analysis

Statistical analyses were performed using the SPSS software (version 24.0; Japan IBM Co., Tokyo, Japan). First, the correlation between each variable and postoperative pneumonia in all 539 patients was analyzed by χ^2^ test and 1-way analysis of variance, followed by multivariate logistic regression analysis. Differences in mean duration of hospitalization and mortality rate between the oral care and control groups were analyzed by Student's *t* test and Fisher's exact test, respectively.

Next, propensity score analysis was performed to minimize selection biases associated with retrospective data analysis between the 2 groups. For each patient, a propensity score for oral care intervention was calculated by logistic regression analysis of all predictive variables. The propensity score matched groups (oral care vs control) were then evaluated by logistic regression analysis for identifying factors associated with the development of postoperative pneumonia. In all analyses, 2-tailed *P* values < 0.05 were considered statistically significant.

### 2.6 Ethics

The study design was approved by the institutional review boards of all participating hospitals. We published research plan and guaranteed opt-out opportunity by the homepage of each hospital.

## 3 Results

Background variables of the original 539 patients in the oral care and control groups before propensity score matching are summarized in Table 1. The tumor stage, frequency of thoracoscopy-assisted esophagectomy, and operation time in the oral care group were greater than those in the control group.

**Table 1:**
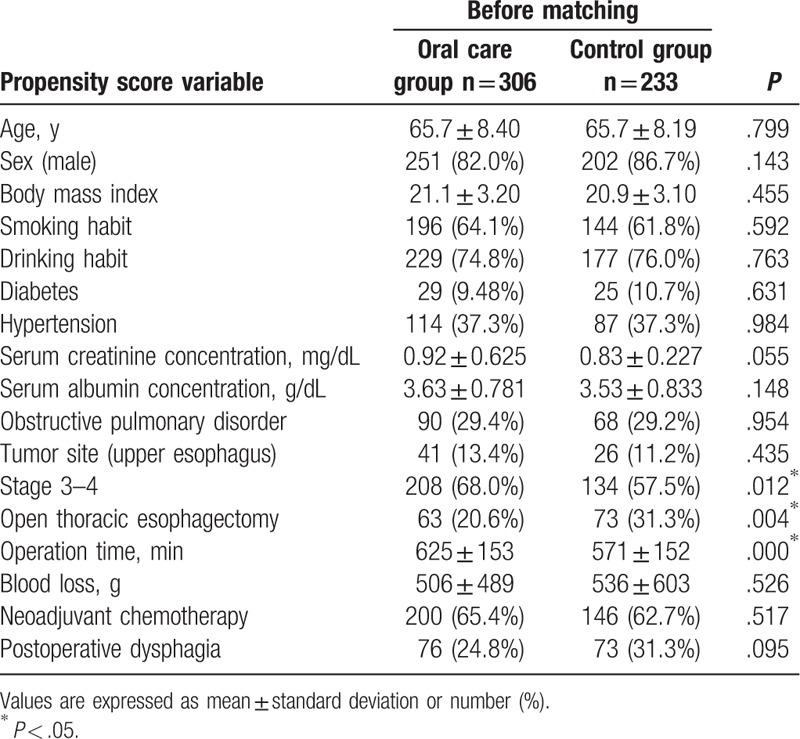
Comparison of variables between the oral care and control groups comprising the original 539 patients.

Of the original 539 patients, 103 (19.1%) experienced postoperative pneumonia, and 3 of 306 patients (0.980%) in the oral care group and 3 of 233 patients (1.29%) in the control group died from complications within 30 days after surgery (*P* = 1.00). There was no significant difference in average duration of hospitalization between the oral care (45.2 days) and control (42.3 days) groups (*P* = .577). Upon univariate analysis, smoking and drinking habits, operation time, postoperative dysphagia, and oral care intervention were found to be significantly related to postoperative pneumonia (Table 2). Of these variables, operation time, postoperative dysphagia, and oral care intervention were found to be significantly correlated with postoperative pneumonia by multivariate analysis (Table 3).

**Table 2:**
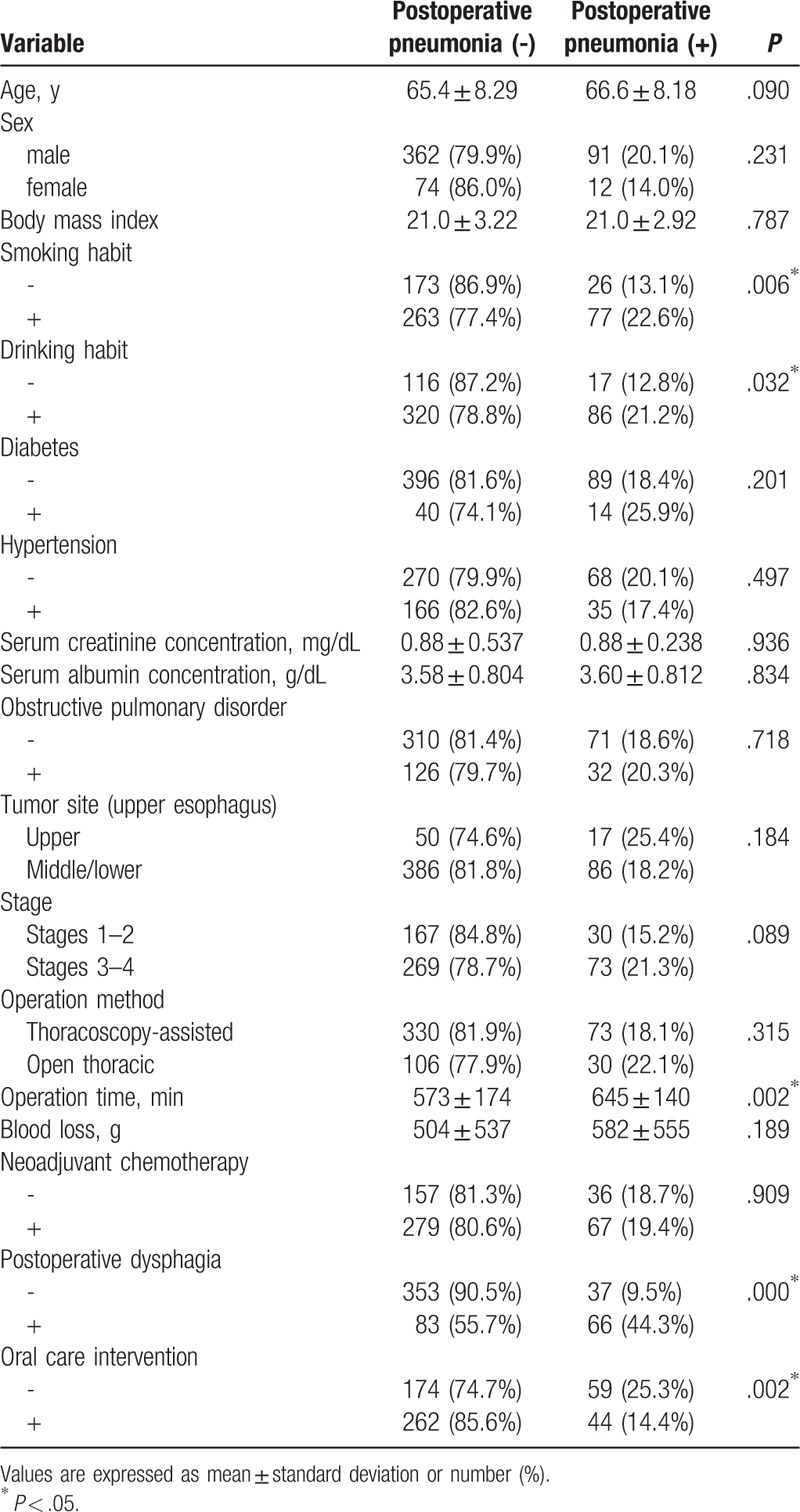
Results of univariate analysis of variables associated with postoperative pneumonia among the original 539 patients.

**Table 3:**
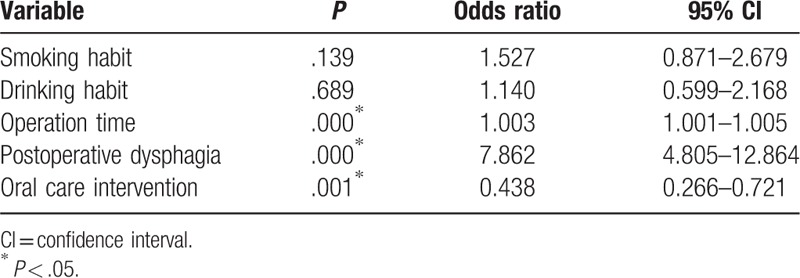
Results of multivariate analysis of variables associated with postoperative pneumonia among the original 539 patients.

Propensity scores were calculated for all patients by logistic regression analysis of all 17 variables associated with oral care intervention. The concordance index (c index) was 0.650—which indicated a strong ability to differentiate between patients with and without oral care—and the Hosmer–Lemeshow statistic was insignificant (*P* = .363), indicating good calibration. Propensity scores—which reflected the probability that a patient would receive oral care intervention—ranged from 0.2704 to 0.9941 in the oral care group and 0.1456 to 0.9117 in the control group.

Propensity score analysis resulted in 420 patients (210 in each group) being matched (Table 4). The results of univariate analysis of the propensity score matched groups revealed old age, smoking and drinking habits, longer operation time, postoperative dysphagia, and lack of oral care intervention to be correlated with development of postoperative pneumonia (Table 5). Table 6 presents the results of multivariate analysis, which revealed 3 significant factors associated with postoperative pneumonia: operation time [*P* = .011; odds ratio (OR), 1.003; 95% confidence interval (95% CI), 1.001–1.005); postoperative dysphagia (*P* = .000; OR, 7.195; 95% CI, 4.084–12.68), and oral care intervention (*P* = .001; OR, 0.365; 95% CI, 0.204–0.653).

**Table 4:**
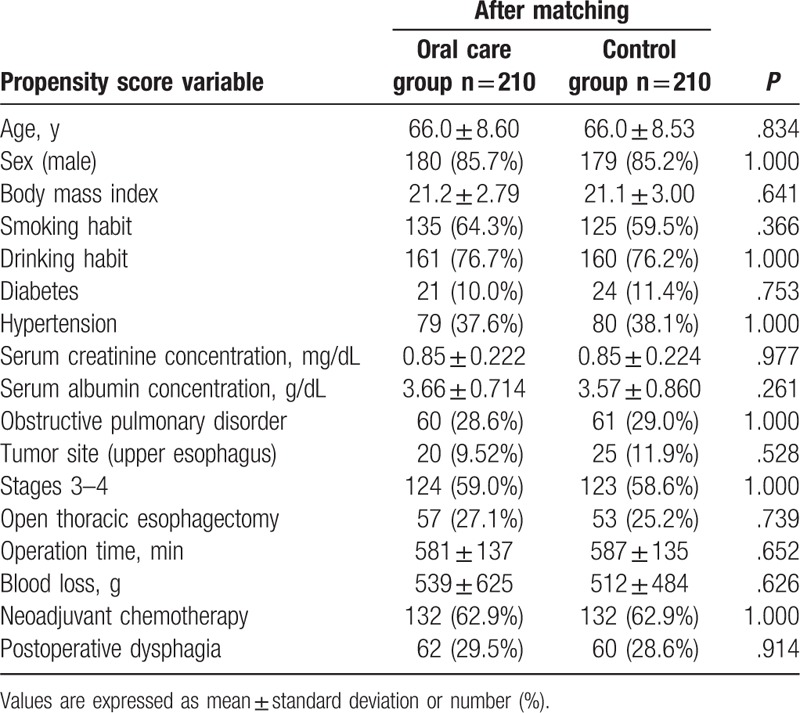
Comparison of variables between the oral care and control groups comprising 420 propensity score matched patients.

**Table 5:**
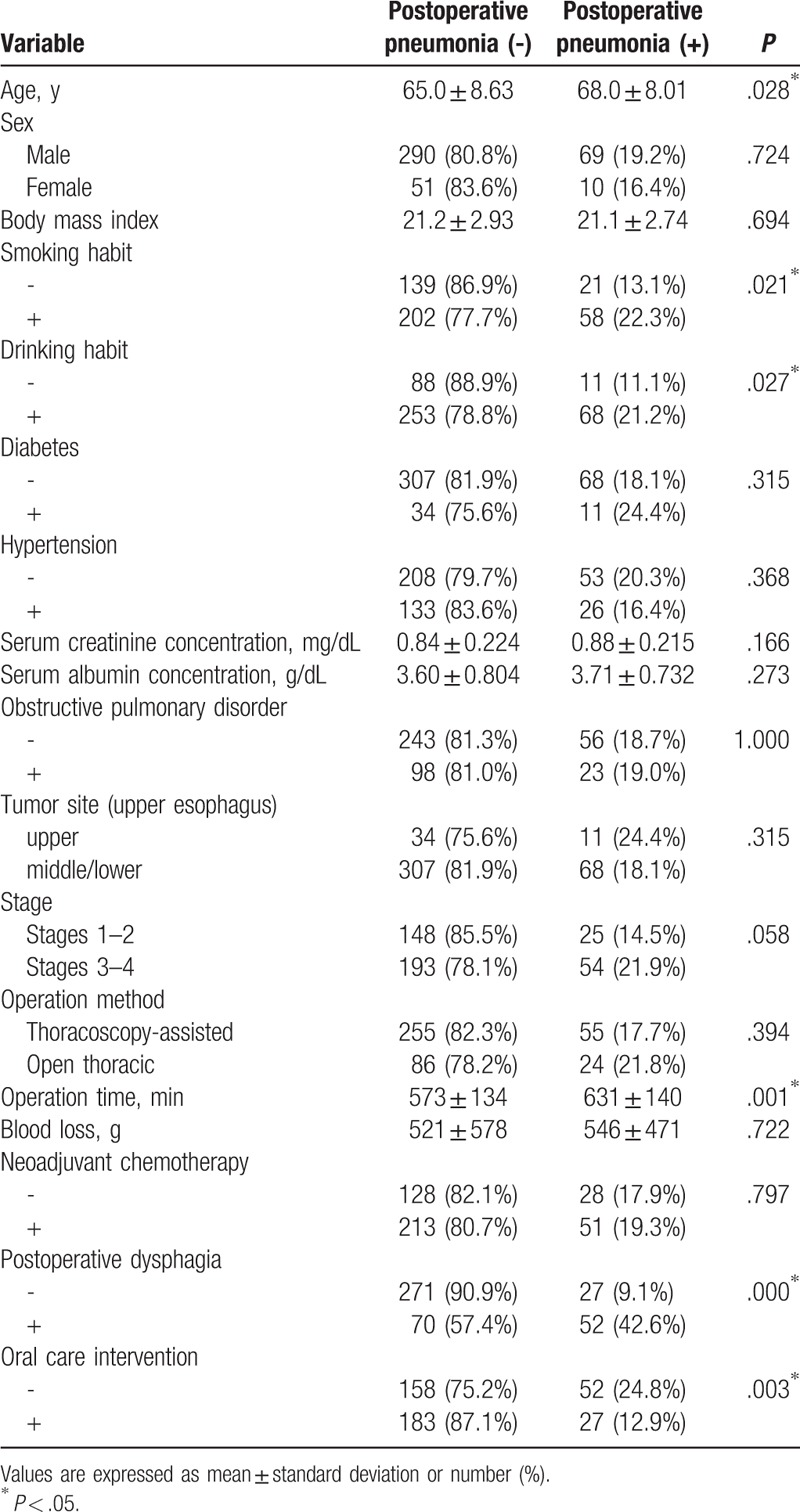
Results of univariate analysis of variables associated with postoperative pneumonia in 420 propensity score matched patients.

**Table 6:**
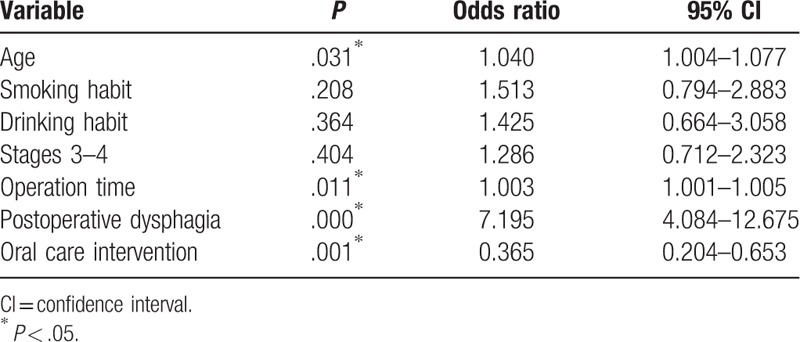
Results of multivariate analysis of variables associated with postoperative pneumonia in 420 propensity score matched patients.

## 4 Discussion

Postoperative pneumonia frequently develops after esophagectomy. It is associated with prolonged hospitalization, higher medical costs, and substantial operative mortality.^[2]^ Previous studies have reported incidence rates of postoperative pneumonia after esophageal surgery as ranging from 7.4% to 50%; in addition, various factors, including regular smoking, decreased pulmonary function, diabetes mellitus, old age, greater surgical stress (operation time, blood loss, and thoracotomy), and general conditions (performance status and complications) have been reported to be correlated with this complication.^[4–18]^ The relatively high incidence of postoperative pneumonia despite the recent advances in surgical procedures and antibiotic therapy indicates the need for new preventive measures.

Dental clearance before major esophageal surgeries is required nowadays, although there have been no studies with high evidence level that showed the effect of oral health care on the prevention of pneumonia after esophageal cancer surgery. Guideline for Diagnosis and Treatment of Esophageal Cancer by The Japan Esophageal Society^[25]^ recommended oral care before surgery to reduce rate of postoperative pneumonia, while in the revised version of 2017,^[26]^ the description of oral care was deleted because of the lack of evidence supporting the efficacy of oral care on the prevention of postoperative pneumonia.

In the present study, 539 patients from 7 hospitals were enrolled, and propensity score analysis was performed to reduce selection biases associated with retrospective data analysis. The incidence rate of postoperative pneumonia after esophageal cancer surgery in the present study (19.1%) corresponded to that reported in previous studies.^[4–18]^ There was no difference in duration of hospitalization between the oral care and control groups, which might be attributable to the fact that duration of hospitalization was affected by other factors, such as adjuvant radiotherapy or chemotherapy. Upon multivariate analysis of the original 539 patients, longer operation time, postoperative dysphagia, and lack of oral care intervention were found to be significantly associated with development of postoperative pneumonia, with postoperative dysphagia exhibiting the strongest correlation with occurrence of pneumonia.

In a statistical analysis of observational data, propensity score matching is a technique that attempts to estimate the effect of a treatment, policy, or other intervention by accounting for the covariates that predict the receipt of treatment.^[27]^ Propensity score matching reduces bias due to confounding variables, which is commonly observed in the estimation of treatment effect by mere comparison of outcomes among units with and without treatment. In the present study, logistic regression analysis of the 420 propensity score matched patients revealed old age, longer operation time, postoperative dysphagia, and lack of oral care intervention to be significantly associated with occurrence of postoperative pneumonia. To the best of our knowledge, this is the first study demonstrating the effectiveness of perioperative oral care in prevention of postoperative pneumonia after esophagectomy with a high evidence level. Further, we believe that our study has generalizability and applicability because the results were obtained with a large number of participants by multicenter analysis.

However, this study has several weaknesses. First, because this is a retrospective study, there is a possibility of unknown confounding factors despite propensity matching analysis. Second, as the 7 hospitals do not have a unified oral care protocol, it is not clear which of the procedures was effective in prevention of postoperative pneumonia. Because oral care has been covered by the Japanese medical insurance system since 2012, and most Japanese patients now receive oral care before surgery, it would be challenging to conduct a randomized controlled trial regarding the preventive effect of oral care. We are planning another multicenter prospective observational study to address these issues.
